# Clinical Presentation and Surgical Management of Five Pediatric Cases with Bronchogenic Cysts: Retrospective Case Series

**DOI:** 10.3390/children9121824

**Published:** 2022-11-25

**Authors:** Ali Alsuheel Asseri, Ayed A. Shati, Amal Y. Moshebah, Omair M. Alshahrani, Rayan M. Saad, Abdulmohsin M. Alzuhari, Maraam M. Al Qout, Abdullah S. Al-Helal

**Affiliations:** 1 Department of Child Health, King Khalid University, Abha 62529, Saudi Arabia; 2 Departments of Pediatric, Abha Maternity and Children’s Hospital, Abha 333042, Saudi Arabia; 3 College of Medicine, King Khalid University, Abha 62529, Saudi Arabia; 4 Departments of Pediatric Surgery, Abha Maternity and Children’s Hospital, Abha 333042, Saudi Arabia

**Keywords:** bronchogenic cyst, lung abscess, complicated pneumonia, mediastinal mass

## Abstract

Background: Bronchogenic cysts (BCs) refer to congenital lesions that result from primitive or abnormal foregut budding, and can be pulmonary or mediastinal. Their occurrence can take place at any point on the tracheobronchial tree, but they are usually localized in the lung parenchyma and mediastinum, and may be symptomatic or asymptomatic. Bronchogenic cyst symptoms can vary, depending on the size and location of the cyst. Methods: A retrospective review of the charts of five patients with a histopathological diagnosis of bronchogenic cysts was performed between 2014 and 2020. The patients reported in this study were diagnosed and managed at Abha Maternity and Children Hospital, Abha, southwest Saudi Arabia. In addition, demographic information, as well as diagnostic and therapeutic information, was provided for each patient, both at discharge and after discharge. All patients had confirmed congenital bronchogenic cysts with different clinical phenotypes and radiological findings. Results: All patients had histopathologically confirmed bronchogenic cysts with different clinical and radiological presentations. Two patients had mediastinal-located cysts; one had a laryngeal cyst; and the last two patients had infected intrapulmonary bronchogenic cysts. All patients underwent complete excision and did not experience recurrence or other postoperative complications during the follow-up period. The latter two patients required lobectomies of the right middle and upper lobes. Conclusions: Although bronchogenic cysts are considered a rare congenital pulmonary malformation, they should be considered in the differential diagnosis of pediatric patients with unusual airway and parenchymal lung manifestations, particularly, persistent stridor, feeding difficulty, and complicated pneumonia. Surgical excision of the cyst is the gold-standard therapy for symptomatic bronchogenic cysts and is highly recommended for asymptomatic ones. Long-term follow-up studies will be required to explore any long-term complications of BCs, particularly regarding the malignancy transformation.

## 1. Introduction

Bronchogenic cysts (BCs) refer to congenital lesions that result from primitive or abnormal foregut budding, which may be pulmonary or mediastinal. Their occurrence can take place at any point on the tracheobronchial tree, but they are usually localized in the lung parenchyma and mediastinum [1,2]. According to Wagner et al. [3], the origin of most BCs can be traced to the mediastinum, whereas only 15–21% of cases indicate their occurrence in the lung parenchyma. According to prior research, BCs make up 10% of all mediastinal masses and are most frequent in males. As a result, mediastinal BCs frequently cause compressive symptoms of the tracheobronchial tree in children [3,4,5,6].

A BC may be symptomatic or asymptomatic. The associated symptoms can vary, depending on the cyst’s size and location, but frequently include a persistent cough, chest pain, increased stridor while sleeping, and swallowing difficulty [2,4,7,8,9]. A frequent complication—especially in cysts with bronchial connections—is infection. The cyst’s rupture could affect the pericardial cavity, the pleural cavity, or the trachea. Pleuritis frequently occurs alongside pneumothorax, which is a common consequence [1,6].

The treatment depends on the symptoms of the disease and the patient’s age. Surgery is most recommended, even for those with the asymptomatic illness. In addition, video-based thoracoscopy is one of the evolving procedures for BC removal that uses the three-port method. Thoracoscopy is advantageous as it reduces pain, scarring, and hospitalization rates [10]. Bronchogenic cysts that are complicated require thoracoscopy. Wedge resection, pericystectomy, or segmentectomy are carried out in patients with peripheral bronchogenic cysts. However, these procedures of mediastinal bronchogenic cyst removal can cause incomplete resection. To prevent recurrence and fluid accumulation, the mucosa needs to be destroyed. Bronchogenic cysts can also be managed through close surveillance imaging in order to ensure that stability is achieved, even if it is temporary [11].

In this study, we discuss five pediatric patients with histopathologically confirmed bronchogenic cysts and a variety of clinical presentations, including aspiration pneumonia, critical airway obstruction, severe respiratory distress with suspected left lung congenital lobar emphysema, an infected right middle lobe bronchogenic cyst, and an infected right upper lobe bronchogenic cyst in cases 1, 2, 3, 4, and 5, respectively.

## 2. Materials and Methods

### 2.1. Study Design, Setting, and Population

A retrospective review of five patients’ charts with a histopathological diagnosis of bronchogenic cysts was performed between 2014 and 2020. The patients reported in this study were diagnosed and managed at Abha Maternity and Children Hospital (AMCH), Abha, southwest Saudi Arabia. Demographic information, as well as diagnostic and therapeutic information (i.e., history, physical examinations, radiological testing, histopathological reports, and follow-up visits), was provided for each patient at discharge and after discharge, as presented in Table 1 and Table 2. This study was approved by the Institutional Review Board at AMCH (REA# 08-07-2022), and written consent was obtained from the patients’ families and documented in their medical records prior to the creation of this report. The diagnostic challenges and surgical interventions were discussed for the enrolled patients.

### 2.2. Diagnosis and Surgical Intervention

All patients had confirmed congenital BCs with different clinical phenotypes and radiological findings. The histopathological diagnosis of a BC includes the presence of a cystic mass containing mucus and ciliary lined with columnar bronchial epithelium. All patients underwent surgical excision of the cyst, and two patients required lobectomy as well, given the intraparenchymal presence of their cysts. All patients were treated with antibiotics preoperatively, until the signs of infections improved, and were then evaluated by the pediatric surgeon for cyst mass removal. None of the enrolled patients had associated congenital anomalies or significant postoperative complications. The pediatric pulmonologist, together with the pediatric surgery team, continued to follow-up with the patients three to four weeks after discharge and four to six months later. In each visit, a full clinical examination was performed and chest imaging was evaluated.

## 3. Results

**Figure 1:**
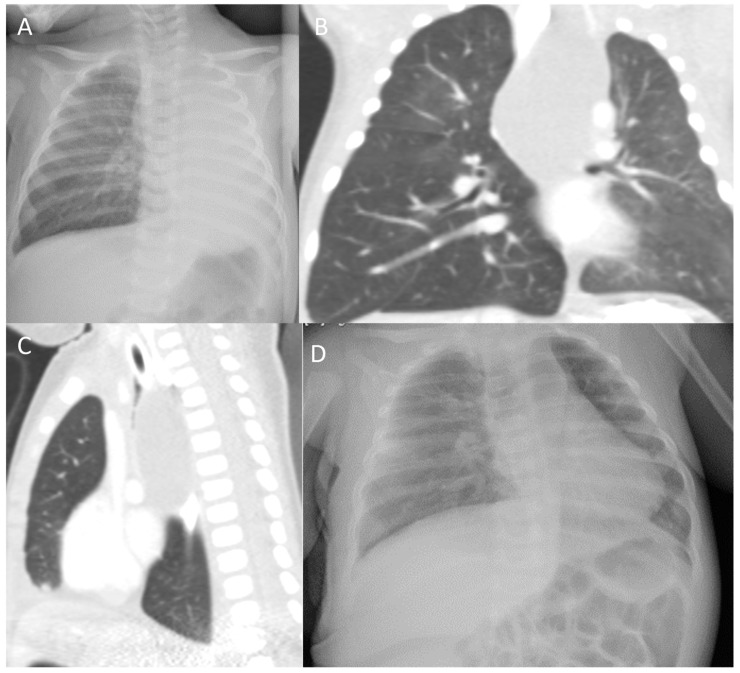
Chest X-ray (CXR) and chest computed tomography (CT) findings in case 1: (**A**) CXR of the patient on admission shows right lung hyperlucency, complete collapse on the left side, and marked left-sided mediastinal shift. (**B**,**C**) Coronal and sagittal windows, lung view; CT images of the chest reveal a well-defined cystic lesion in the posterior mediastinum at the level of the left apical lobe extending downward to the level below the carina with fluid density. The mass lesion attaches to the adjacent vessels and compresses the aortic arch, the descending aorta to the left side, and the inferior vena cava to the right side. The lesion shifts the esophagus to the right side with no obvious communication. (**D**) CXR of the patient at 1-month postoperative follow-up showing a well-aerated lung.

Case 1: A 1-month-old boy presented to the pediatric emergency room (ER) with recurrent episodes of choking and cough since his second week of life. The parents reported that the symptoms occurred immediately after feeding. In addition, they reported progressively increasing labored breathing over a week prior to the ER presentation. There was no fever or contact with sick patients at home. The patient was delivered at full term and had an uneventful postnatal course. Initial physical examination revealed that the patient was tachypneic with a respiratory rate of 70 breaths/minute and costal retractions. His oxygen saturation was 84% on room air, and he was afebrile. Chest examination revealed bilaterally decreased air entry with right-side expiratory wheezes; the rest of his physical examination was normal. A chest X-ray (CXR) was taken for abnormal breath sounds and revealed right lung hyperlucency, complete collapse on the left side, and a marked left-sided mediastinal shift (Figure 1A). A chest computed tomography (CT) scan showed a well-defined cystic lesion in the posterior mediastinum that extended downward to the level below the carina, causing a compression effect with significant narrowing of the carina and the left and right main bronchi (more on the left side), while causing the trachea to shift to the right side. The mass lesion was attached to the adjacent vessels and compressed the aortic arch, the descending aorta to the left, and the inferior vena cava to the right. In addition, the lesion shifted the esophagus to the right side, with no obvious communication (Figure 1B,C). These results supported the diagnosis of a bronchogenic cyst in the posterior mediastinum. As a result, the patient underwent a thoracotomy and the cystic mass was surgically removed. Pathology revealed a cystic lesion lined with ciliated columnar epithelium and surrounded by a fibromuscular wall containing cartilage and nests of bronchial submucosal glands, confirming the diagnosis of a bronchogenic cyst. The postoperative course was uneventful. A CXR was repeated a month later, which revealed a well-aerated lung without any consolidations or collapse (Figure 1D). The patient is now seven years old and free of respiratory symptoms and signs, with normal growth and development.

Case 2: A 6-month-old boy presented to the ER after a week of progressively increasing labored breathing. The mother stated that the patient had had noisy breathing since birth, which worsened after the patient’s recent upper respiratory infection. Physical examination revealed severe respiratory distress, inspiratory stridor, and diminished air entry over the lung fields. A CXR was immediately obtained, which showed hyperinflated lungs with clear lung fields. The patient was intubated and ventilated due to critical respiratory distress, which did not improve with high-flow oxygen. A CT scan of the chest and neck subsequently demonstrated a large neck mass compressing the central airways and causing critical obstruction; the impression was a bronchogenic cyst. The patient then underwent laparoscopic-assisted mass removal. He was hospitalized for eight days and then discharged with a normal medical condition. The histopathology report revealed a cyst lined by pseudostratified ciliated epithelium with areas of squamous metaplasia, and there was no evidence of malignancy. The cyst wall contained smooth muscle and hyaline cartilage. The final histopathological conclusion was a mediastinal bronchogenic cyst. Subsequently, the patient experienced recurrent episodes of viral-induced wheezing and was diagnosed with mild asthma. He is currently 4 years old and is on a low-dose inhaled corticosteroid; his asthma is well-controlled.

**Figure 2:**
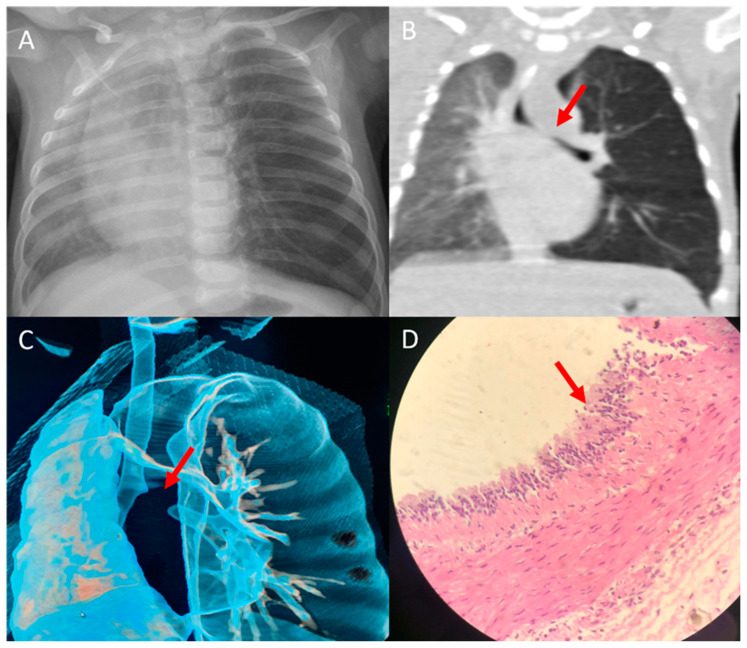
Chest X-ray (CXR) and chest computed tomography (CT) findings in case 3: (**A**) CXR of the patient on admission shows severe hyperinflation on the left side. (**B**) CT scan of the chest shows a hypodense cystic lesion in the middle mediastinum (arrow), causing compression on the distal trachea and the proximal aspect of the left mainstem bronchi, with resultant hyperinflation of the left lung. (**C**) Three-dimensional reconstruction of the CT scan shows a complete obstruction of the left main bronchus (arrow), caused by external compression of the adjacent fluid-filled cystic lesion. (**D**) Photomicrograph showing the cyst lined by ciliated pseudostratified columnar cells with a variable number of goblet cells filled with serous material. The cyst wall contains smooth muscle fibers with no granuloma or malignant cells (H&E, ×100 magnification).

Case 3: A 3-month-old girl presented with cough, fever, and shortness of breath for three days. Examination upon arrival at the ER revealed that the patient had tachypneic with grunting and intercostal and subcostal retractions. Her oxygen saturation was 80% on room air. A CXR showed a hyperinflated left lung with right upper lobe opacity (Figure 2A). A CT scan of the chest revealed a 3.3 cm × 2.9 cm × 2.4 cm well defined hypodense cystic lesion, at the level of the carina (Figure 2B). There was also a mass effect involving the left mainstem bronchus (Figure 2B,C), with resultant hyperinflation of the left lung. The patient was admitted to the pediatric intensive care unit and connected to a high-flow nasal cannula (HFNC). In addition, she was started on broad-spectrum antibiotics. A nasopharyngeal real-time polymerase chain reaction viral respiratory panel was performed and negative results were reported. In addition, the blood culture was negative. After seven days of hospitalization and improvement in her clinical condition (weaned off HFNC and afebrile), the pediatric surgery team was consulted, and they removed the mediastinal mass through a thoracoscopic excision. During the procedure, the cyst was found to be attached to the surrounding structure and was precisely removed. Microscopical evaluation of the cyst confirmed the diagnosis of a bronchogenic cyst, which revealed that the cystic wall was lined with ciliated columnar or pseudostratified columnar epithelium, and the walls contained cartilaginous islands (Figure 2D). Postoperatively, the patient had an uncomplicated course and had no signs of respiratory decompensation. The patient is two years old, has no chronic respiratory symptoms, and is growing normally.

**Figure 3:**
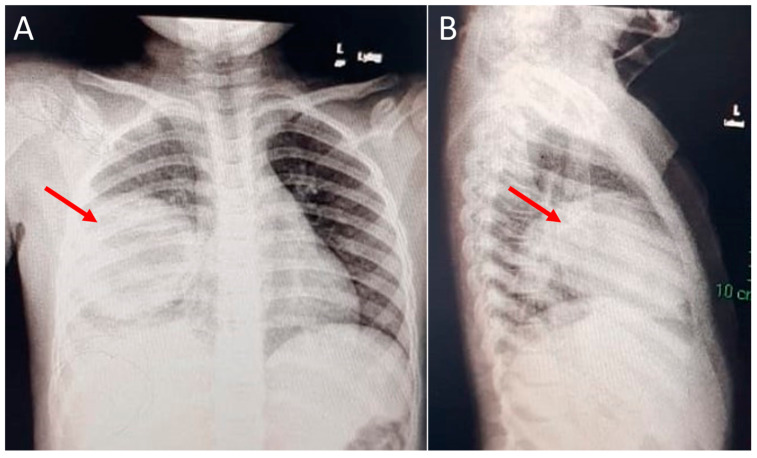
Chest X-ray (CXR) findings in case 4: (**A**) Anterior-posterior CXR view of the patient on admission shows a large, well-defined, homogenous opacity, rounded in shape, occupying the right middle lobe and silhouetting the right cardiac border; no definitive air bronchogram or cavitary changes were seen within the lesion (arrow). (**B**) Lateral CXR view of the patient shows the mass localized to the right middle lobe and encroaching upon the right lower lobe (arrow).

Case 4: A 60-month-old girl presented with a 3-day history of shortness of breath and 5 days of high-grade fever. A chest radiograph showed a consolidated lesion occupying the right middle lobe (Figure 3 A,B), and a chest CT scan revealed a well-circumscribed, rounded, 90 × 70 mm-diameter cyst on the right middle lobe, with an air bronchogram associated with necrotizing characteristics and a central abscess measuring 6 cm at its greatest diameter. The patient was admitted and started on intravenous antibiotics, which alleviated the symptoms partially. Hence, pediatric surgery was consulted, and the patient underwent a lobectomy of the right middle lobe. Microscopic examination of the cyst revealed that the cyst wall was mainly composed of fibrous tissue with abundant chronic inflammatory and hemorrhagic cells. In addition, the cyst wall was partly lined with stratified squamous epithelium. The histopathological findings were consistent with an infected bronchogenic cyst. The patient was discharged eight days after resection and has remained in a stable condition.

**Figure 4:**
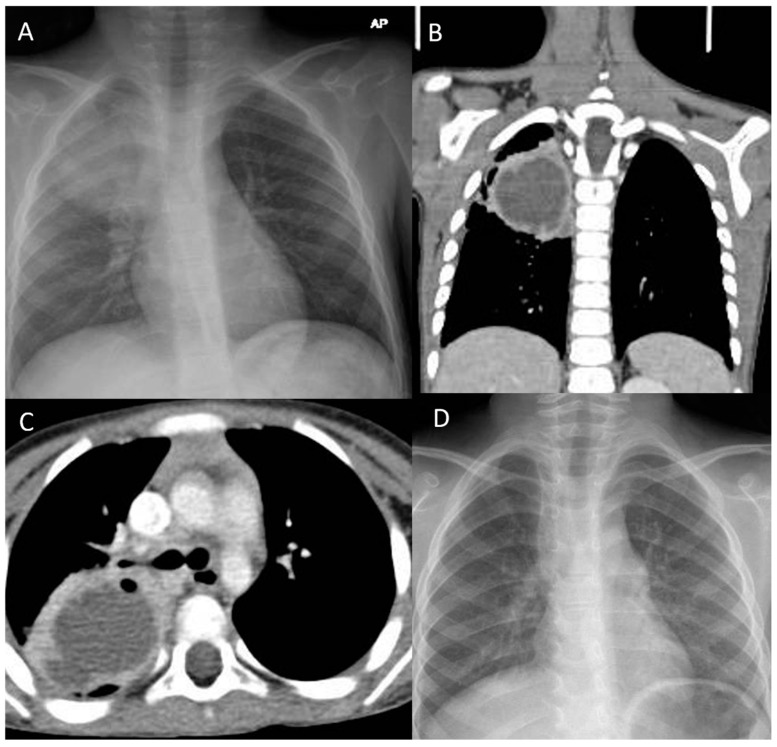
Chest X-ray (CXR) and chest computed tomography (CT) findings in case 5: (**A**) CXR of the patient on admission shows opacification involving the right upper lobe. (**B**,**C**) Coronal and axial windows, mediastinal view; CT scan of the chest shows a large, thick-walled cavity filled with fluids with heterogeneous opacity occupying the right upper lobe, leading to pleural and chest wall adhesions. (**D**) CXR of the patient at the 5-month postoperative follow-up shows well-aerated lung without any consolidations or air leakage.

Case 5: A 72-month-old boy presented with a history of 8 weeks of high-grade fever, wet cough, and weight loss. He had an unremarkable neonatal and medical history. The family denied a history of traveling or contact with a pulmonary tuberculosis patient. Prior to the presentation to the ER, the patient received multiple courses of parenteral and oral antibiotics without improvement. Upon arrival, he had a high temperature (39.5 °C), a respiratory rate of 34 breaths/min, a heart rate of 132/min, a blood pressure of 110/65 mmHg, and an oxygen saturation of 90% on room air. The initial examination revealed that the patient was undernourished and had mild respiratory distress. Chest examination revealed decreased air entry over the right upper zone with inspiratory crackles. Notable laboratory findings on admission revealed a white blood cell count of 16.34 k/µL and a predominance of neutrophils at 11.9 k/µL, whereas lymphocytes were measured at 2.94 k/µL; Hb, 10.3 g/dl; platelets, 372 k/µL; C-reactive protein, 32 mg/L (<10 mg/L); and the erythrocyte sedimentation rate, 95 mm/h (0–20 mm/h). In addition, the blood culture was negative, and electrolytes, and renal and liver function were normal. The test for human immunodeficiency virus was negative. The CXR showed a consolidated lesion occupying the right upper lobe (Figure 4A). The patient was admitted and started on broad-spectrum antibiotics (ceftriaxone, vancomycin, and clindamycin). The professional diagnosis was likely complicated pneumonia with a right upper lobe abscess, with likely underlying right upper lobe intraparenchymal bronchogenic cysts. Three early morning gastric aspirates were negative for *Mycobacterium tuberculosis* DNA. A chest CT scan was ordered, which revealed a large, thick-walled cavity filled with fluids and with heterogeneous opacity occupying the right upper lobe (Figure 4B,C). The final diagnosis was right upper lobe intraparenchymal bronchogenic cysts complicated by abscess formation. The broad-spectrum IV antibiotics were continued until the patient’s symptoms improved with normalization of the acute-phase inflammatory markers. The pediatric surgery team was consulted and proceeded with lobectomy of the right upper lobe. The patient had a smooth course of surgery with no complications. Histopathology examination revealed that the cyst was lined by ciliated pseudostratified columnar cells with a variable number of goblet cells filled with serous material. The cyst wall contained smooth muscle fibers with no granuloma or malignant cells. The clinical, imaging, and histopathological findings were consistent with intraparenchymal bronchogenic cysts. The patient stayed two weeks in the hospital and was then discharged home in good condition. A CXR was repeated five months postoperatively, which revealed a well-aerated lung without any consolidations or air leakage (Figure 4D).

## 4. Discussion

In this study, we describe five pediatric patients with histopathologically confirmed bronchogenic cysts. Two patients had mediastinal cysts, and one patient had a laryngeal cyst. The remaining two patients had infected intrapulmonary cysts. Beginning in the embryo, there is a complex interaction between the foregut and the airway. The aerodigestive organs are formed from nearby foregut segments in the original foregut. The primitive foreguts and/or their mesenchyme are the common ancestor of the airway and lung buds, the throat, the esophagus, the stomach, and the diaphragm, which all have overlapping regulatory mechanisms [1,2].

Esophageal symptoms (particularly feeding problems), critical airway obstruction, and severe lower respiratory symptoms (cough, fever, dyspnea, and hypoxia) were the most common clinical presentations in the enrolled patients, in line with previously reported case reports and case series [2,3,4,7,8,11,12,13,14]. In cases 1 and 3, the patients presented with recurrent cough and choking since the second week of life, and dynamic airway compression mimicking congenital lobar emphysema (CLE). The team diagnosed the patients with mediastinal BCs, which were surgically removed, and the diagnosis of BC was confirmed histopathologically. Due to the severe left main-stem bronchus in case 3, the diagnosis of CLE was suspected. Compressive symptoms of BCs could cause severe dynamic airway obstruction masquerading as CLE. Therefore, careful radiological evaluation (particularly chest CT) is mandatory prior to surgical interventions. For instance, Arun et al. reported a 3-day-old neonate who had severe respiratory distress, where the plain chest X-ray revealed CLE. Prior to the surgery, a chest CT scan was performed, which revealed a BC compressing the main-stem bronchi, causing severe air trapping and mimicking the CLE diagnosis [13].

Although the exact prevalence of BCs among children is unknown, several studies have estimated that its estimated prevalence among congenital pulmonary malformation is 1:42,000–1:68,000 [4,15]. Mediastinal BCs are the most common, representing around 85% of cases [16]. On the other hand, parenchymal cysts represent only approximately 15% of cases [8].

Stridor is an unusual, high-pitched sound that occurs when airflow is turbulent through an airway that is partially blocked at the supraglottis, glottis, subglottis, or trachea level. Case 2 involved a 6-month-old child who presented with critical airway obstruction. A CT scan of the chest and neck subsequently demonstrated a large neck mass compressing the central airways and causing critical obstruction; the impression was a bronchogenic cyst. A laryngeal BC should be considered in the differential diagnosis of infants with persistent stridor and critical airway obstruction. Lu et al. reported a single case report of a 12-year-old child who presented with chronic hoarseness and progressively increasing labored breathing, who was later diagnosed with a laryngeal BC and required surgical excision [5]. Furthermore, Xu et al. reported 13 cases of congenital BCs with various clinical and radiological presentations. In their case series, one case had early neonatal respiratory distress and required intubation. A laryngopharyngeal mass was identified, and the patient underwent surgical removal of the mass. Later, it was histopathologically confirmed as a BC [17]. The proposed mechanism of the laryngeal cyst causing critical airway obstruction is that the mass effect of the fluid-filled cyst causes the lumen to narrow and significant turbulence flow.

Cases 4 and 5 presented with high-grade fever, cough, and labored breathing. The initial CXRs revealed right middle and upper lobe consolidations, respectively. Case 4 was diagnosed with a right middle lobe abscess, whereas case 5 had a right upper lobe lung abscess. The patients underwent lobectomies, and the cysts were histopathologically confirmed as BCs. While most intrapulmonary BCs are located in the lower lobes, several case series have reported BCs involving the upper lobes, masquerading as hydatid cysts or mycobacterium infections [16,18].

The timing and severity of the symptoms can vary, according to the location, size, and complications of the cyst. The symptoms of infected cysts and severe compressive symptoms have been described in several published case series [3,5,7,16]. Imaging is recommended in order to identify the site and size of the cyst. Although CT is a helpful diagnostic modality to identify the site and characteristics of the cyst, the associated diagnostic accuracy is around 50% [7]. A recently evolving diagnostic modality is magnetic resonance imaging, which can often depict spherical cystic masses with varying signals based on the characteristics of the cysts and the presence of infection [17].

Complete surgical excision with detailed histopathological study of the excised cyst—whether mediastinal or pulmonary—is the gold-standard therapy. Intrapulmonary lesions may require segmental or lobar excision. Elective cyst excision is recommended, before the compression of normal structures results in critical respiratory distress and the need for emergency excision [18]. The time of the surgery depends on the symptomatology. In addition, the removal of asymptomatic cysts is somewhat controversial. Due to the risk of infections, compression of vital organs and, seldomly, malignancy transformation, many experts recommend surgical excision [1,18].

This study had a few limitations. Drawing a generalizable conclusion from this study is difficult, as it involved only five patients. In addition, a lack of antenatal diagnosis and regular postnatal follow-up led to an inability to identify the exact cyst size at birth and the cyst measurements before they became symptomatic. The patients’ lack of long-term follow-up is another limiting factor that could have helped to assess the physiological assessment of pulmonary function and the natural course of the respiratory illness.

## 5. Conclusions

Although bronchogenic cysts are considered a rare congenital pulmonary malformation, they should be considered in the differential diagnosis of pediatric patients present with unusual airway and parenchymal lung manifestations, particularly persistent stridor, feeding difficulty, and complicated pneumonia. Surgical excision of the cyst is considered the gold-standard therapy for symptomatic bronchogenic cysts, and is also highly recommended for asymptomatic ones. Long-term follow-up studies will be required to explore any long-term complications of BCs, particularly regarding malignancy transformation.

## Figures and Tables

**Table 1:** Clinical characteristics of the patients.

Clinical Features	Case 1	Case 2	Case 3	Case 4	Case 5
Age in months/sex	1/M	6/M	3/F	60/F	72/M
Antenatal diagnosis	−	−	−	+	−
Age at first symptom appearance (months)	1	2	1	60	72
Presenting symptoms and signs					
- Fever	−	+	+	+	+
- Cough	+	+	+	+	+
- Shortness of breath	+	+	+	+	+
- Upper airway obstruction	−	+	−	−	−
- Dysphagia	+	+	+	−	−
- Hypoxia	+	+	+	+	+
Pulmonary involvement					
- Lung collapse	+	−	−	−	−
- Hyperinflated lung	−	+	+	−	−
- Pneumonia	+	−	+	+	+
Cyst characteristics					
- Unilocular	+	+	+	+	+
- Evidence of infection	+	−	−	+	+
- Rupture	−	−	−	−	−

Present (+); absent (−).

**Table 2:** Cyst location, surgical procedures, operative findings, and complications.

Clinical Features	Case 1	Case 2	Case 3	Case 4	Case 5
Location					
- Posterior mediastinum	+	−	+	−	−
- Larynx	−	+	−	−	−
- Lung parenchyma	−	−	−	+(RML)	+(RUL)
Surgical procedures					
- Lobectomy	−	−	−	+	+
- Excision	+	+	+	−	−
Operative findings					
- Cyst adhesion to adjacent tissues	+	+	+	+	+
- Pleural adhesion	−	−	−	+	+
Complications					
- Intraoperative	−	−	−	−	−
- Postoperative	−	+	−	+	+

Right middle lobe (RML); right upper lobe (RUL). Present (+); absent (−).

## Data Availability

The data sets used in this study are available from the corresponding authors upon request.

## References

[1] Aktoǧu S., Yuncu G., Halilçolar H., Ermete S., Buduneli T. (1996). Bronchogenic cysts: Clinicopathological presentation and treatment. Eur. Respir. J..

[2] McAdams H.P., Kirejczyk W.M., Rosado-de-Christenson M.L., Matsumoto S. (2000). Bronchogenic cyst: Imaging features with clinical and histopathologic correlation. Radiology.

[3] Wagner M.L., Hart C.K., Benscoter D., Fleck R.J., Tiao G.M. (2020). Congenital lung overinflation secondary to a unilateral obstructing mediastinal bronchogenic cyst. J. Pediatr. Surg. Case Rep..

[4] Abushahin A., Zarroug A., Wagdi M., Janahi I. (2018). Bronchogenic Cyst as an Unusual Cause of a Persistent Cough and Wheeze in Children: A Case Report and Literature Review. Case Rep. Pediatr..

[5] Lu D., Yu R., Yang H., Liu J. (2017). A bronchogenic cyst of the larynx: A case report. Exp. Ther. Med..

[6] Han C., Lin R., Yu J., Zhang Q., Zhang Y., Liu J., Ding Z., Hou X. (2016). A case report of esophageal bronchogenic cyst and review of the literature with an emphasis on endoscopic ultrasonography appearance. Medicine.

[7] Gursoy S., Ucvet A., Ozturk A.A., Erbaycu A.E., Basok O., Yucel N. (2009). Seven years experience of bronchogenic cysts. Saudi Med. J..

[8] Cohn J.E., Rethy K., Prasad R., Mae Pascasio J., Annunzio K., Zwillenberg S. (2020). Pediatric Bronchogenic Cysts: A Case Series of Six Patients Highlighting Diagnosis and Management. J. Investig. Surg..

[9] Lim L.L., Ho K.Y., Goh P.M. (2002). Preoperative diagnosis of a paraesophageal bronchogenic cyst using endosonography. Ann. Thorac. Surg..

[10] Lesaffer J., Heremans B., De Leyn P., Van Raemdonck D. (2011). Video-assisted mediastinoscopic resection of a large symptomatic bronchogenic cyst. Interact. Cardiovasc. Thorac. Surg..

[11] Diraneyya O.M., Al-Mutrafi A., Al Jadaan S., Eldadah O., Alghamdi M.H., Alghamdi A.A., Alhabshan F. (2021). Bronchogenic cyst presenting with respiratory distress in a neonate. Cardiol. Young.

[12] Brcic I. (2018). Bronchogenic Cyst. Mediastinal.

[13] Arun S., Kumar M., Ross B.J. (2016). Mediastinal bronchogenic cyst mimicking congenital lobar emphysema. BMJ Case Rep..

[14] Khemiri M., Ouederni M., Mansour F.B., Barsaoui S. (2008). Bronchogenic cyst: An uncommon cause of congenital lobar emphysema. Respir. Med..

[15] Coselli M.P., De Ipolyi P., Bloss R.S., Diaz R.F., Fitzgerald J.B. (1987). Bronchogenic cysts above and below the diaphragm: Report of eight cases. Ann. Thorac. Surg..

[16] Sarper A., Ayten A., Golbasi I., Demircan A., Isin E. (2003). Bronchogenic cyst. Tex. Heart Inst. J..

[17] Xu Y., Han F., Seng D., Jiang L., Wang S., Ni X., Zhang J. (2021). A Clinical Analysis of Pharyngeal Bronchogenic Cysts in the Pharynx of Children. Front. Pediatr..

[18] Suen H.C., Mathisen D.J., Grillo H.C., LeBlanc J., McLoud T.C., Moncure A.C., Hilgenberg A.D. (1993). Surgical management and radiological characteristics of bronchogenic cysts. Ann. Thorac. Surg..

